# Primary Open Angle Glaucoma and Post-LASIK Keratectasia

**Published:** 2010-07

**Authors:** Mohammad-Reza Razeghinejad, Kouros Nouri-Mahdavi, Shamira Perera

**Affiliations:** Poostchi Ophthalmology Research Center, Shiraz University of Medical Sciences, Shiraz, Iran; Glaucoma Division, Jules Stein Eye Institute, University of California, Los Angeles, USA; Consultant, Glaucoma Service, Singapore National Eye Center, Singapore

## CASE PRESENTATION

The patient presented herein is a 52-year-old woman suffering from primary open angle glaucoma (POAG) in both eyes. She has no history of systemic disorders and is not on any systemic medications. She underwent laser in situ keratomileusis (LASIK) 9 years ago for refractive error of −5.50−3.00×180 in both eyes, and was diagnosed with glaucoma 7 years afterwards. Her ocular examination when I saw her for the first time two years ago was as follows.

**Figure 1:**
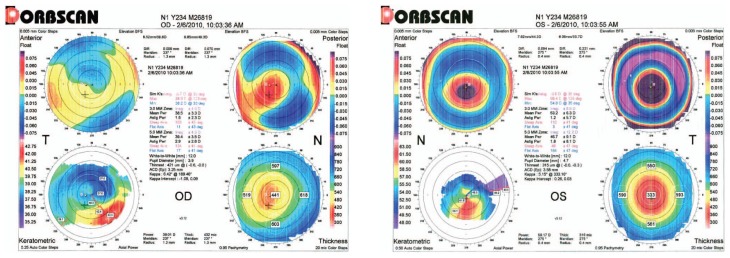
Orbscan printouts of the patient.

**Figure 2:**
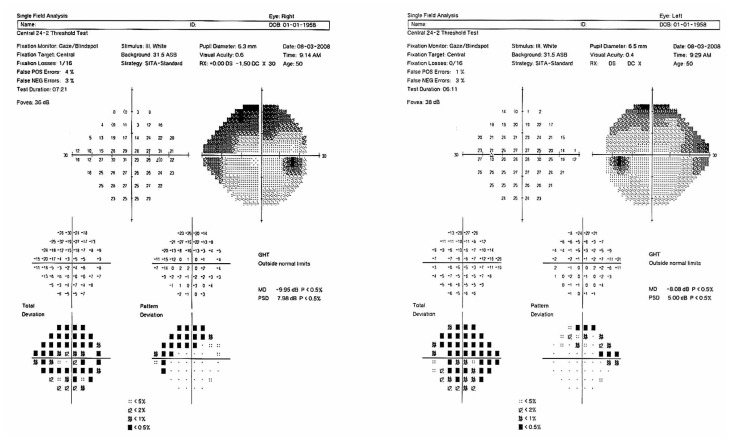
Baseline perimetry.

Best corrected visual acuity (BCVA) was 6/10 and 3/10 in the right and left eyes with −4.00−1.00×30 and −13.00−5.50×180 respectively while wearing rigid gas permeable (RGP) contact lenses. Slitlamp examination revealed a LASIK flap and signs of corneal ectasia in the left eye, mild nuclear sclerosis changes were evident in both eyes and other slitlamp findings were unremarkable. Orbscan images (Orbscan II, Bausch & Lomb, Salt Lake City, USA) are shown in figure 1. Gonioscopy revealed widely open anterior chamber angles with no synechiae. Intraocular pressure (IOP) by Goldmann applanation tonometry (GAT) was 10 and 12 mmHg in the right and left eyes respectively while receiving latanoprost (once daily), timolol (twice daily) and dorzolamide (twice daily) in both eyes. Central corneal thickness (CCT) measured 431 and 322 microns in her right and left eyes respectively. Fundus examination revealed average-sized discs with vertical cup to disc ratios of 0.9 and 0.8 in the right and left eyes respectively, together with inferior rim loss; the macula, vessels and periphery were unremarkable. Baseline automated perimetry (Humphrey Field Analyzer II, Humphrey Systems, Carl Zeiss Meditec Inc., Dublin, USA) is shown in figure 2.

**Figure 3:**
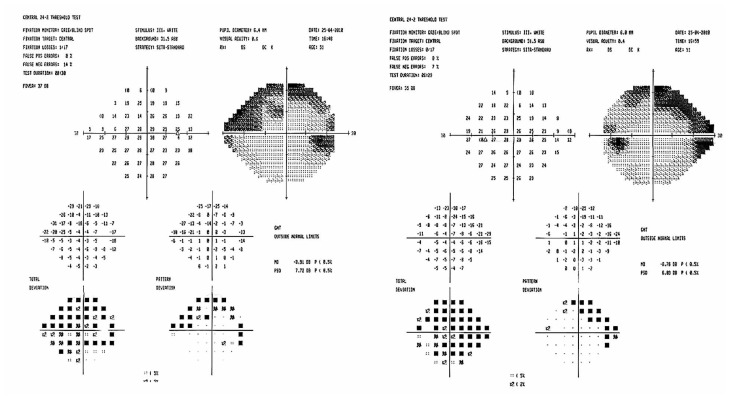
Follow-up perimetry after 2 years.

**Figure 4:**
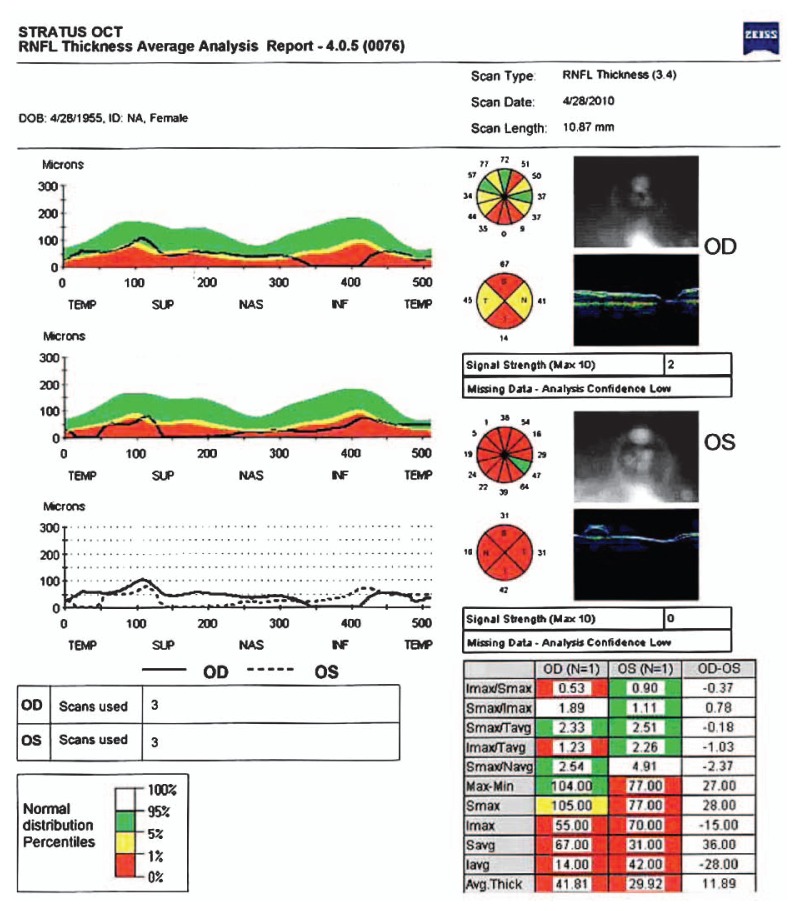
Stratus OCT printout.

**Figure 5:**
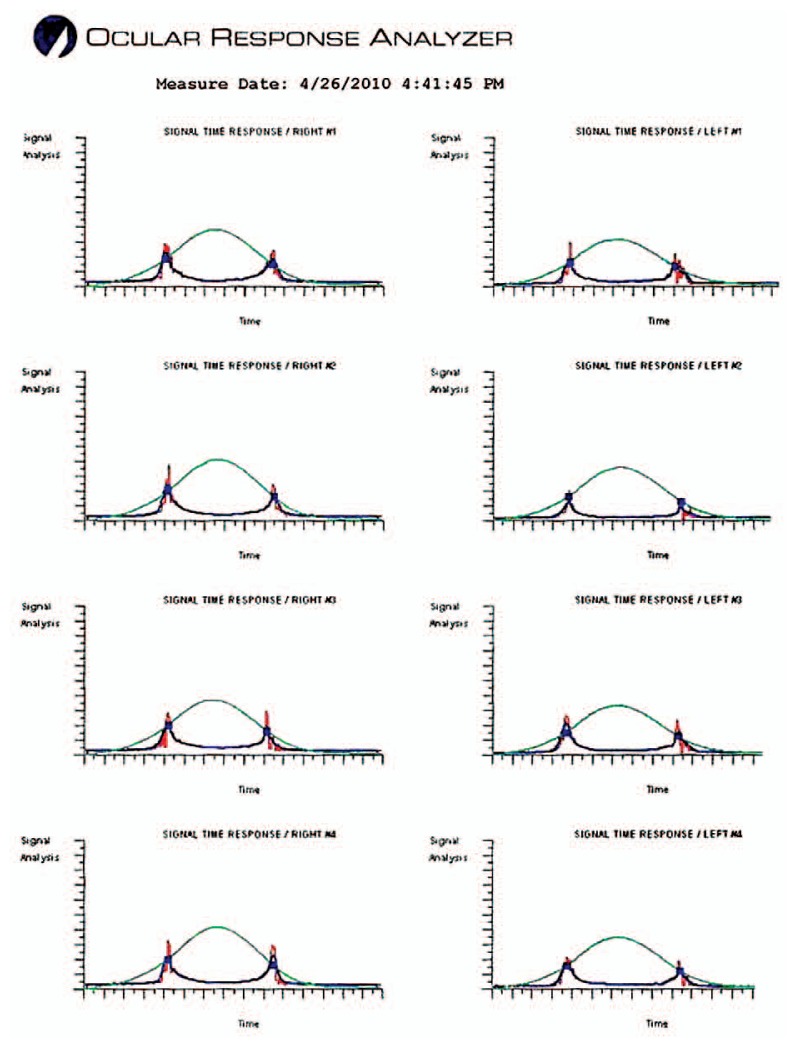
ORA printout at last follow-up.

Considering that target IOP had been achieved, I suggested that she be followed closely with medications. She was observed for two years, but on her last examination I noticed suspicious progression of cupping and visual field defects especially in her left eye. The follow-up visual field and Stratus OCT (Carl Zeiss Meditec, Dublin, USA) scans of the peripapillary nerve fiber layer are shown in figures 3 and 4. At this time, IOP was measured by the ocular response analyzer (ORA) (Reichert Ophthalmic Instruments, Buffalo, USA) which reported cornea-compensated IOP (IOPcc) of 22 and 21 mmHg in the right and left eyes, respectively (Fig. 5).

Herein we present the opinions of three glaucoma specialists on the diagnosis and management of this patient.

### Mohammad-Reza Razeghinejad, MD

This 52-year old lady is a case of POAG with history of prior LASIK surgery. IOP is acceptable while measured with GAT, but high with the ORA. Clinical and visual field evaluation show signs of glaucoma progression.

The patient has a thin cornea (OD: 431 and OS: 322 microns) and IOP measured with GAT should be corrected on the basis of corneal thickness. The Goldmann tonometer is assumed to measure IOP accurately when CCT is 520 microns and many correction formulas have been introduced for thicker and thinner corneas.^1^ Although all reports agree that CCT affects IOP measurement, there is no consensus regarding a specific formula for IOP correction in routine clinical practice. The suggested formulas do not seem to be precise in patients with very thin or thick corneas, such as this patient. Moreover, it is not clear whether any specific conversion factor can be applied to patients who have undergone corneal refractive surgery.^1^ It has been shown that corneal hysteresis (CH) and corneal resistance factor (CRF) are decreased in eyes with previous keratorefractive surgery.^2^ The only available instrument that can measure these factors and correct the measured IOP accordingly is the ORA. In this patient, no data are provided about CH and CRF, but considering the significant difference between IOPcc values determined by ORA and GAT readings, they should be low. Some studies suggest using the Dynamic Contour Tonometer (Pascal tonometer) in patients with previous corneal refractive surgery, but because of mixed results, it is hard to select one instrument as the device of choice. In a recent study, IOP was measured with Goldmann and Schiotz tonometers before and after LASIK surgery in 23 eyes. Interestingly, Schiotz measurements were consistent before and after surgery while Goldmann readings were significantly lower after LASIK.^3^ With respect to the available data, I would prefer to rely on IOPs measured with the ORA in this patient.

The baseline perimetry is reliable and shows generalized depression and typical superior arcuate visual field defects. The cause of generalized suppression can be the cataracts or refractive error (possibly, the refractive error had not been corrected while performing the visual field, because there is no data about the refraction in the printout). In the follow-up perimetry, defect depth has increased and progression of the visual field defect is obvious. In the OCT, the circle is not centered on the optic nerve and signal strengths are 2 and 0 in the right and left eyes, respectively. Currently, for most studies, the minimum acceptable signal strength is six.^4^ With respect to the cornea and cataract, it seems impossible to obtain pictures of higher quality. To me, recording the optic nerve head findings and obtaining serial visual fields are appropriate tools for following this patient.

Due to corneal ectasia in the left eye and regression of myopia in the right eye, the patient wears RGP contact lenses. A portion of this myopia may be due to nuclear sclerosis. It has been demonstrated that prostaglandins can cause corneal thinning^5^, and although the reported effects are not severe, I would prefer not to use this class of medications in this particular patient. The mentioned effect has been attributed to PGF_2_-induced up-regulation of matrix metalloproteinases with subsequent alterations of the extracellular matrix of the corneal stroma. These effects may eventually lead to corneal weakening.^6^ A suitable alternative can be brimonidine, though its efficacy is not as much as latanoprost and it entails a higher rate of ocular allergy.

Since acceptable target IOP cannot be attained with medical therapy and the patient is already on 3 topical drugs, a laser or surgical intervention is necessary. I would try laser trabeculoplasty and if this is ineffective, the next step would be incisional surgery. The gold standard surgery for patients with primary open angle glaucoma is trabeculectomy. In this patient because of dependence on contact lenses, a blebless surgery is recommended to prevent bleb associated infections. Viscocanalostomy which is regarded as a blebless procedure leads to bleb formation in 5% of all patients, according to Stegmann et al.^7^ Moreover, in about 50% of patients who undergo viscocanalostomy, laser goniopuncture is necessary which will convert the procedure to a penetrating intervention and may produce a bleb. Post-viscocanalostomy IOPs are usually in the high teens which would not be an acceptable level in this patient. Trabectome is another option but similar to viscocanalostomy, it may not reduce IOP to the low teens. Moreover, this patient may require corneal transplantation in the future or simultaneously with glaucoma surgery. Because of the low grade post-transplant inflammation, the possibility of trabeculectomy bleb fibrosis is high. Based on the available literature, the preferred surgery in grafted patients seems to be a shunt. All in all, I prefer shunt surgery if laser trabeculoplasty is not effective. In contrast to the prior literature, IOPs in the low teens are achievable with shunts, as was attained in 62% of patients in the tube group at 3 years in the “Trabeculectomy versus Tube” trial.^8^

Suggested Readings1BashfordKPShafranovGTauberSShieldsMBConsiderations of glaucoma in patients undergoing corneal refractive surgerySurv Ophthalmol2005502452511585081310.1016/j.survophthal.2005.02.0062PeposeJSFeigenbaumSKQaziMASandersonJPRobertsCJChanges in corneal biomechanics and intraocular pressure following LASIK using static, dynamic, and noncontact tonometryAm J Ophthalmol200714339471718804110.1016/j.ajo.2006.09.0363CronembergerSGuimaraesCSCalixtoNCalixtoJMIntraocular pressure and ocular rigidity after LASIKArq Bras Oftalmol2009724394431982078010.1590/s0004-274920090004000034ChangRBudenzDLNew developments in optical coherence tomography for glaucomaCurr Opin Ophthalmol2008191271351830128610.1097/ICU.0b013e3282f36cdf5HatanakaMVessaniRMEliasIRMoritaCSusannaRJrThe effect of prostaglandin analogs and prostamide on central corneal thicknessJ Ocul Pharmacol Ther20092551531923201410.1089/jop.2007.01256NowroozzadehMHProstaglandin analogs may aggravate myopic regression after laser in situ keratomileusisAm J Ophthalmol20091477547551932744610.1016/j.ajo.2008.12.0137StegmannRPienaarAMillerDViscocanalostomy for open-angle glaucoma in black African patientsJ Cataract Refract Surg1999253163221007943510.1016/s0886-3350(99)80078-98GeddeSJSchiffmanJCFeuerWJHerndonLWBrandtJDBudenzDLTube Versus Trabeculectomy Study GroupThree-year follow-up of the tube versus trabeculectomy studyAm J Ophthalmol20091486706841967472910.1016/j.ajo.2009.06.018

### Kouros Nouri-Mahdavi, MD, MSc

This is a 52-year old Caucasian female who was diagnosed with glaucoma, 7 years after LASIK. In the mean time, she has also developed post-LASIK ectasia with corneal thinning, more marked in the left eye along with worsening of myopia and induced astigmatism, again much worse in the left eye. Pressures have been seemingly well controlled in both eyes with the caveat that the real IOPs are actually unknown especially in the left eye where CCT is extremely thin. ORA measurements confirm a significant underestimation of IOP in both eyes with waveforms typical of keratoconus in both eyes. There is now strong suspicion of progression at the level of the visual fields in both eyes and the disc in the left eye, although no baseline images are available to compare to. Stratus OCT images obtained at final follow-up are unfortunately not helpful, given the very low signal strengths and poor segmentation of RNFL in both eyes.

I think the first thing to establish is whether the visual field progression is real. If repeat testing confirms progression in the left eye, then the rate of progression seems fast enough to warrant aggressive treatment, given the patient’s young age and her life expectancy. Nonsurgical options are limited. Brimonidine is rarely tolerated on a long-term basis and is not likely to have much of an effect as the fourth medication. The same is true with laser trabeculoplasty. Unless the patient is reluctant to have surgery, I would recommend trabeculectomy with mitomycin C, since the newer angle surgeries are unlikely to achieve the low target pressure required in this patient. Of course, it would be very difficult to titrate the endpoint in such a case postoperatively: avoiding hypotony and maculopathy in a young myopic patient and at the same time reaching a low enough target IOP. Multiple tight sutures should be placed with gradual laser suture lysis postoperatively to prevent severe hypotony. Checking the pressure postoperatively with Dynamic Contour Tonometry or ORA may also be helpful. I would aim for a target of around 5–6 mmHg based on GAT readings, although I should admit that this is only a rough guess.

For the right eye, in which progression is still not quite established, I would step up treatment by doing a selective laser trabeculoplasty and adding brimonidine to reach a target in the range of 6–8 mmHg based on GAT and then, closely watch the patient for evidence of progression and results of surgery in the left eye. She may need surgery soon in the right eye as well.

### Shamira Perera, MBBS, BSc, FRCOphth

This case outlines a scenario where a post-LASIK eye exhibits keratectasia and glaucoma progression, likely due to underestimated IOP by GAT. It highlights the intricacies of glaucoma management in myopes with astigmatism and previous LASIK.

The assessment of glaucoma in highly myopic eyes is fraught with difficulty. The optic discs are notoriously difficult to assess as they have large diameters, greater cup disc ratios, and shallower optic cups. The single unreliable (with poor signal strength) Stratus OCT scan (Fig. 4) adds little to this difficult diagnosis as the absolute values for retinal nerve fiber thickness are often globally reduced in high myopes. Visual fields are also difficult to judge, as myopic retinal degeneration and tilted discs may mimic glaucomatous visual field defects. Hence, it is possible that glaucoma may have been present and missed in this case prior to LASIK.

Accurate IOP measurement is crucial for monitoring glaucoma in such cases. There is 5.5 D of astigmatism in the left eye and ideally, GAT should be performed in two perpendicular directions and averaged. GAT is inaccurate when there is high astigmatism or deviation from CCT of 500 μm. Studies have shown a 2 to 7 mmHg underestimation of IOP by GAT per 100 μm of corneal thinning. The ORA and Dynamic Contour Tonometer are purported to be independent of changes in CCT and therefore may reflect a closer measure of the actual IOP in this case. It could be advisable to continue the use of the ORA to monitor IOP in the long term for consistency.

As the IOPcc provided by ORA is actually far above target IOP, it is likely that glaucoma progression is IOP related. With the evidence of visual field and optic disc progression in a relatively young individual, trabeculectomy with mitomycin C is the preferred option. Care is needed as the increased axial length, thinner sclera, and larger intraocular volume predispose myopic eyes to hypotony, shallow anterior chambers, and choroidal effusions after surgery.

Difficulties with continuing RGP contact lens use after trabeculectomy are well known and the patient must be counseled that she may need to return to wearing glasses or eventually require corneal surgery for visual rehabilitation.

Suggested Readings1JonasJBDichtlAOptic disc morphology in myopic primary open-angle glaucomaGraefes Arch Clin Exp Ophthalmol1997235627633934994610.1007/BF009469382RauscherFMSekhonNFeuerWJBudenzDLMyopia affects retinal nerve fiber layer measurements as determined by optical coherence tomographyJ Glaucoma2009185015051974566410.1097/IJG.0b013e318193c2bePMC27427643DoughtyMJZamanMLHuman corneal thickness and its impact on intraocular pressure measures: a review and meta-analysis approachSurv Ophthalmol2000443674081073423910.1016/s0039-6257(00)00110-74EhlersNBramsenTSperlingSApplanation tonometry and central corneal thicknessActa Ophthalmol (Copenh)1975533443117291010.1111/j.1755-3768.1975.tb01135.x5KotechaAWhiteESchlottmannPGGarway-HeathDFIntraocular pressure measurement precision with the Goldmann applanation, dynamic contour, and ocular response analyzer tonometersOphthalmology20101177307372012273710.1016/j.ophtha.2009.09.0206ParkHYLeeNYParkCKRisk factors of shallow anterior chamber other than hypotony after Ahmed glaucoma valve implantJ Glaucoma20091844481914213410.1097/IJG.0b013e31816b2fe7

## References

[1] Bashford KP, Shafranov G, Tauber S, Shields MB (2005). Considerations of glaucoma in patients undergoing corneal refractive surgery. Surv Ophthalmol.

[2] Pepose JS, Feigenbaum SK, Qazi MA, Sanderson JP, Roberts CJ (2007). Changes in corneal biomechanics and intraocular pressure following LASIK using static, dynamic, and noncontact tonometry. Am J Ophthalmol.

[3] Cronemberger S, Guimaraes CS, Calixto N, Calixto JM (2009). Intraocular pressure and ocular rigidity after LASIK. Arq Bras Oftalmol.

[4] Chang R, Budenz DL (2008). New developments in optical coherence tomography for glaucoma. Curr Opin Ophthalmol.

[5] Hatanaka M, Vessani RM, Elias IR, Morita C, Susanna R (2009). The effect of prostaglandin analogs and prostamide on central corneal thickness. J Ocul Pharmacol Ther.

[6] Nowroozzadeh MH (2009). Prostaglandin analogs may aggravate myopic regression after laser in situ keratomileusis. Am J Ophthalmol.

[7] Stegmann R, Pienaar A, Miller D (1999). Viscocanalostomy for open-angle glaucoma in black African patients. J Cataract Refract Surg.

[8] Gedde SJ, Schiffman JC, Feuer WJ, Herndon LW, Brandt JD, Budenz DL, Tube Versus Trabeculectomy Study Group (2009). Three-year follow-up of the tube versus trabeculectomy study. Am J Ophthalmol.

